# Experiences with implementing advance care planning (ACP-GP) in Belgian general practice in the context of a cluster RCT: a process evaluation using the RE-AIM framework

**DOI:** 10.1186/s12875-024-02510-5

**Published:** 2024-07-06

**Authors:** Julie Stevens, Anne-Lore Scherrens, Peter Pype, Luc Deliens, Aline De Vleminck, Koen Pardon

**Affiliations:** 1 End-of-Life Care Research Group, Vrije Universiteit Brussel & Ghent University, Laarbeeklaan 103, Brussels, 1090 Belgium; 2 Department of Family Medicine and Chronic Care, Vrije Universiteit Brussel (VUB), Laarbeeklaan 103, Brussels, 1090 Belgium; 3 Department of Public Health and Primary Care, Ghent University, Corneel Heymanslaan 10, Gent, 9000 Belgium

**Keywords:** Advance care planning, General practice, Process evaluation, RE-AIM Framework, Mixed methods, Serious illness, Chronic life-limiting illness

## Abstract

**Background:**

General practice is often recommended as an ideal setting to initiate advance care planning (ACP), but uptake of ACP in this setting is low. ACP-GP is a complex intervention to facilitate ACP for patients with chronic, life-limiting illness in Belgian general practice. It aims to increase patient ACP engagement and general practitioner (GP) ACP self-efficacy. In a cluster-randomized controlled trial, the intervention was not superior to control in increasing these outcomes. A parallel process evaluation aimed to enhance understanding of how the intervention was implemented, and which factors might have influenced trial results.

**Methods:**

We conducted a mixed-methods process evaluation following the Reach, Effectiveness, Adoption, Implementation, Maintenance (RE-AIM) framework. Data sources include recruitment and implementation monitoring, questionnaires for patients and GPs, and semi-structured (focus group) interviews with patients and GPs. Questionnaire data were analyzed descriptively. Qualitative data were first analyzed inductively; themes were then assigned deductively to RE-AIM dimensions.

**Results:**

Thirty-five GPs and 95 patients were recruited to the trial; GP reach was low. Sixteen GPs and 46 patients provided questionnaire data at 3 months post-baseline; qualitative data were transcribed for 14 GPs and 11 patients. Adoption of intervention components was moderate to good, with the exception of the documentation template for GPs. Interviews revealed varying patient attitudes towards ACP, but patients nonetheless emphasized that conversations made them feel reassured. GPs especially valued a positive framing of ACP. When adopted, the intervention was well-implemented and participant satisfaction was high. However, intention for maintenance was moderate, with GPs raising questions of how to sustainably implement ACP conversations in the future.

**Conclusions:**

Implementing the complex ACP-GP intervention in general practice is feasible, and can be successful. However, the implementation process is challenging and the sustainability is suboptimal. Our findings will guide future research and recommendations for facilitating and implementing ACP in general practice.

**Trial registration:**

ISRCTN12995230; prospectively registered on 19/06/2020.

**Supplementary Information:**

The online version contains supplementary material available at 10.1186/s12875-024-02510-5.

## Background

Advance care planning (ACP) is an iterative process whereby people communicate with family, loved ones, and health providers about personal values, life goals, and preferences regarding (future) treatment and care [1]. While ACP should not be limited to patients with chronic, life-limiting illness, it plays a crucial role in providing high-quality care for people with such conditions, supporting decision-making regarding future care [2]. Research suggests that patients and the general population perceive ACP as important [3–6], but uptake remains low, including in general practice [7–9]. These findings conflict with recommendations to introduce ACP in a timely manner, for which general practitioners (GPs) are well-situated. Initiating ACP in general practice allows patients to discuss values and wishes for care at a time when their health is relatively stable [10, 11]. GPs can leverage their longstanding relationship with the patient to facilitate these conversations. Such continuity is seen as an important task of GPs in Belgium, who also liaise with palliative home care. Belgian patients with terminal illness also expect their GP to exchange information with specialist care [12]. However, GPs may face barriers to initiating ACP in practice, such as insufficient skills and a lack of time [13–16].

The ACP-GP intervention was developed to facilitate ACP conversations in Belgian general practice. Following the Medical Research Council (MRC) guidance framework for development of complex interventions [17], barriers and facilitators to ACP in this setting were identified. These included perceived patient factors, such as lack of understanding about ACP; GP factors, such as a lack of confidence and skills to initiate ACP; and system-level factors, such as lack of a place to consistently record patient care wishes [14]. Key intervention components, based on existing literature, were selected to target barriers and support facilitators. The components were refined after expert panel review [18], and further after a pilot study [19]. This yielded the ACP-GP intervention, a complex intervention with four interacting components, which was tested in a cluster-randomized controlled trial (RCT). Briefly, the intervention consisted of (1) GP training in ACP communication; (2) A patient workbook; (3) Two ACP conversations between patient and GP; and (4) A template to document the conversations. (See Additional File 1 for details)

We conducted a cluster-RCT to evaluate whether the intervention was superior to usual care in increasing patient and GP primary outcomes. For patients, we measured ACP engagement with the 15-item ACP Engagement Survey [20]. For GPs, we measured self-efficacy to conduct ACP, using the ACP-Self Efficacy (ACP-SE) scale [21]. At 3 months post-baseline assessment (T1), we found that although outcomes increased in both groups, the intervention group did not increase significantly more than the control group [22].

To open the “black box” of this complex ACP intervention and understand why we observed these outcomes, a thorough process evaluation is necessary [23]. This can aid in distinguishing between problems related to intervention theory, and those associated with intervention delivery [24]. We therefore aim to evaluate experiences with implementation of the intervention, as reported by patients and GPs who participated.

We embedded a process evaluation in the cluster-RCT to enhance our understanding of how the intervention was implemented and interacted with contextual factors, facilitators and barriers encountered during implementation, and how these interacted to influence outcomes. The process evaluation aligns with the (updated) MRC Framework guidance, which emphasizes that complex intervention research can address questions beyond whether the intended outcome is achieved, e.g. by identifying other impacts and assessing the value of the intervention, in light of resource demands [25].

## Methods

### Design

This process evaluation follows the Reach, Efficacy/Effectiveness, Adoption, Implementation, Maintenance (RE-AIM) framework [26]. This framework allows researchers to evaluate how and why an intervention works (or not) when implemented in health system settings [27]. It is an intuitive model of evaluation which can be used to consider pragmatic questions of “Who, what, where, how, and when?” to understand key findings when evaluating the intervention [28]. The addition of qualitative assessment in this design also allows exploration of “Why?”, such as by exploring factors influencing adoption or implementation, to complement the above [29].

We conducted this mixed-methods process evaluation, starting from the beginning of recruitment and ending after the 6-month intervention period. We use a sequential design, with quantitative data collection during, and qualitative data collection after, the intervention period [30]. RE-AIM informed the conduct, analysis, and structure of this manuscript. The conceptualization of the RE-AIM dimensions and corresponding data collection are shown in Table 1.

**Table 1:** RE-AIM dimensions, operationalization, and measurements used for the present study

RE-AIM dimension	Operationalization	Measurement
Reach The number, proportion, and representativeness of participants in the study	• Number of GPs and patients identified • Number of GPs and patients who agreed to participate • Comparing participants with non-participants	• Documentation of the recruitment process by the researchers • Documentation of reasons given for not participating • Participant demographics
Effectiveness The impact of the intervention, including potential negative effects	• Primary and secondary RCT outcomes • Adverse events	• Questionnaires at T0, T1, T2 • Reports of any adverse events
Adoption (individual level) The extent of uptake of intervention components by participants, and factors affecting this: • Are decisions made to engage with the intervention? To what extent? • What contributes to these decisions?	• GP attendance at the training • GP use of documentation templates • Patient use of the work booklet • Experiences of GPs and patients applying intervention components (workbook, conversations, documentation) (e.g. reasons for (not) applying, changes in GP practice)	• Training checklist (after each training) • Questionnaire for GPs regarding their ACP practices and conversations in the last 3 months (T1) • Questionnaire for patients regarding ACP conversations with their GP in the last 3 months (T1) • Review of documentation template use via questionnaire and copies returned to the researchers (physical copy or digital scan) (T1, T2) • Contents of work booklet from a sample of patients in the intervention group (physical copy or digital scan) (T1, T2) • Focus groups with GPs (after T2) • Semi-structured interviews with patients (after T2)
Implementation (individual level) The extent to which the intervention was implemented as intended, satisfaction with the intervention, and factors affecting this • How was the intervention carried out? • What hindered or helped participants in carrying out the intervention?	• Fidelity: the extent to which the steps of the intervention were followed as specified in the protocol • Satisfaction of GPs and patients with the intervention components • Patient and GP barriers/facilitators encountered while implementing components of the intervention	• Training checklist (after each training) • Review of documentation template use via questionnaire and copies returned to the researchers (physical copy or digital scan) (T1, T2) • Satisfaction questionnaire for intervention GPs and patients (T1): Items asking about usefulness of, and satisfaction with, the intervention (Questions used a Likert scale (e.g., “How useful did you find the conversations with your GP, based on the workbook?”, response range 1–7, 1 = Not at all useful, 7 = Very useful) or categorical answers (e.g., “To what extent did the conversations with your GP, based on the workbook, meet your expectations?”; answers options “They did not meet my expectation”, “They met my expectations”, “They exceeded my expectations”.) • Focus groups with GPs (after T2) • Semi-structured interviews with patients (after T2)
Maintenance The intention to sustain the intervention over time, and how the intervention can be improved for the future	• GP intention for using the intervention materials in the future • Recommendations by the GP and patients to improve intervention usability in the future	• Satisfaction questionnaires for intervention GPs and patients: item asking about interest to use the intervention in the future (T1) • Focus groups with GPs (after T2) • Semi-structured interviews with patients (after T2)

### Setting and participants

Participants were recruited in the scope of the cluster-RCT of the ACP-GP intervention in Belgian general practice. Eligible for participation were Belgian GPs and their Dutch-speaking patients with chronic, life-limiting illness (advanced/unresectable cancer, organ failure, frailty), for whom the GP would not be surprised if they were to die within the next 12–24 months. In group GP practices, one GP per practice was included. For more information about the cluster-RCT design, we refer to the published protocol [31].

### Data collection

During recruitment, a trial manager and data collectors maintained records of participants contacted and noted reasons for declining participation. Participants completed demographics questionnaires at baseline (T0). The trial manager and data collectors also monitored for adverse events.

All participating GPs and patients were asked to complete questionnaires about their ACP conversations and satisfaction with the intervention, using a self-developed satisfaction questionnaire, at T1, 3 months post-baseline. This timing was chosen because primary effectiveness was measured at T1.

We conducted semi-structured interviews with intervention participants in March-June of 2021. Interview guides with open questions and probes guided data collection (Additional File 2). As we aimed to encourage discussion between GPs about their experiences, we invited GPs to focus groups. If attendance was not feasible, individual interviews were possible. Focus groups were moderated by JS, ADV, and an assisting researcher, and conducted via video conferencing due to COVID-19 restrictions. JS and an assisting researcher individually interviewed a convenience sample of patients by telephone. We interviewed patients individually due to practical constraints and to avoid overburdening patients. Focus groups and interviews were audio-recorded; if recording was not possible, extensive written notes were taken. Recordings were transcribed verbatim and pseudonymized.

### Analysis

Questionnaire data were analyzed descriptively in SPSS software (Version 27). To ease interpretation, 7-point Likert scales were reduced to three categories (1–3: low rating or disagreement; 4: neutral rating; 5–7: high rating or agreement).

Qualitative data analyses were based on a content analysis approach, combining inductive and deductive analysis [32]. First, JS and A-LS independently read and inductively coded a selection of transcripts. During meetings, the two authors checked similarities and differences in coding and interpretation before coming to an agreement about a preliminary coding structure. Two coding trees were established, for patients and GPs respectively. JS coded the remaining transcripts in NVivo software (Version 12). Overarching themes were grouped deductively, linking them to the RE-AIM framework dimensions. JS, A-LS, ADV, and KP, held meetings to review the coding structure and achieve consensus about interpretation of key findings.

## Results

18 GPs and 53 patients were assigned to the intervention. Sixteen GPs and 46 patients returned questionnaires at T1. After the intervention period, we conducted three focus groups (*n* = 3, *n* = 2, *n* = 5 GPs respectively), and interviewed four GPs individually. Thirteen patients from the intervention group were interviewed. One recording of a patient dyad (married partners both participating in the intervention, interviewed simultaneously) was inaudible and not transcribed, yielding 11 patient transcriptions. Demographics of interviewed participants are show in Table S1 (Additional File 3).

For the qualitative reporting of results, we note that factors affecting adoption (participants making the decision to initiate intervention components) and implementation (how the adopted components are carried out in practice) were often interconnected. Results should be read with this in mind. All qualitative themes and illustrative quotes are shown in Table 2.

**Table 2:** Factors influencing each RE-AIM dimension, as reported by GPs and patients during interviews and focus groups

	Factors reported by			
	GPs	Illustrative GP quote	Patients	Illustrative patient quote
RE-AIM dimension				
Reach	Limitations of the inclusion criteria	*“It’s complex*,* but I think there were a few interesting patients we could have included*,* if French and English were included as languages for consultation.” (Quote GP1.1 Focus group 3)*		
	Varying usefulness of the surprise question	*“For one patient*,* I thought: they really need it. But for the other two*,* it’s possible that they pass away but I could see them living another five years as well. But I thought it was needed.” (Quote GP1.2 GP interview 2)*		
	Some patients with chronic life-limiting illness are not seen by the GP until they approach the terminal phase	*“I think it’s especially the people who always see a specialist. Some cancer patients you don’t see for a whole year*,* but they are monitored by a specialist. I think we miss them. When they have exhausted their treatment options*,* then they come to us.” (Quote GP1.3 Focus group 1)*		
	Selection bias by GPs	*“But in the group that meets [the criteria]*,* you choose the people you’ve known for longer or with whom you feel comfortable. I would never have asked it of someone I have only seen once in my practice*,* even if they met the criteria. These are people with whom you feel comfortable*,* and you know the patient is also comfortable with you.” (Quote GP1.4*,* GP interview 4)*		
Effectiveness	Increased GP intention to take initiative in ACP conversations	*“I would maybe start it myself*,* before I would have waited until a patient came to me with something. Now I’ll talk about it myself*,* even in situations that aren’t urgent*,* as I just said*,* because you do it anyway*,* A bit*,* a year ahead of time*,* opening up that conversation.” (Quote GP2.1*,* Focus Group 1)*	Facilitates patients reflecting about future health, values, and wishes	*Patient: “You get a different take on things*,* take on life a little bit. So…”* ***Interviewer: “Yes?”*** *Patient: “You start seeing it differently.”* ***Interviewer: “Yes***,*** and in what way?”*** *Patient: “Yes*,* what could happen. Or what you’ll be confronted with. That*,* that*,* I wouldn’t think about that otherwise*,* now you think about that.” (Quote PT2.1*,* Patient interview 11)*
	More positive framing of ACP	*“What happened to me especially*,* is that the stories in that workbook*,* the tendency and the tone of the stories*,* the positive approach. It’s had an enormous impact. I will take that with me for the rest of my career.” (Quote GP2.2*,* Focus Group 2)*	Wishes are documented and communicated to family	*“Well*,* Dr. [name] made a list together with me […] of what I would and would not want. Every child received that on their computer. So now everyone is aware of the situation*,* of what I would want.” (Quote PT2.2*,* Patient interview 10)*
	ACP process is facilitated: GPs learn and new and useful information about patients’ experiences and values, document outcomes of ACP conversations	*“The experience with illness and dying in their surrounding environment was good to hear*,* because there were things there that I didn’t know. They are people I don’t follow up for 20 years*,* I’ve worked in the practice for five years. It’s useful to hear things that also give you insight into why they do or don’t want certain things.” (Quote GP2.3*,* GP Interview 1)*	Positive affective outcomes	***Interviewer: “Yes***,*** so that was the value for you***,*** that it’s all on paper now.”*** *Patient: “Yes. That’s a big reassurance for me.”* ***Interviewer: “Yes***,*** yes. So you feel reassured that***,*** uh…”*** *Patient: “That I can count on her if something happens*,* yes.” (Quote PT2.3*,* Patient interview 11)*
	GPs feel capable to speak up for patient wishes and values	*“In the meantime*,* I’ve been able to apply that a few times*,* and express it to the family for example. Someone who is palliative and unable to speak anymore*,* if you can express it that way*,* you notice it brings about a sense of peace: That’s true*,* our dad… Then everyone is at peace with it and they stand behind your approach.” (Quote GP2.4*,* Focus group 1)*		
	Perception that patients actively contemplate ACP	*“I had two conversations*,* one with just the patient and during the second one I also spoke with the daughter. Those were very useful conversations*,* where the patient also said: ‘I’m glad I did this. I also actively considered it.’” (Quote GP2.5*,* Focus group 3)*		
	Previous experience influences whether outcomes change	*“My feeling of being prepared did not change much. Because I actually had that already*,* since I conducted many conversations for my thesis. That’s mainly building confidence in yourself.” (Quote GP2.6*,* GP interview 2)*		
Adoption	GPs feel that ACP, while a delicate subject, is important to do	*“[Referring to the goals of ACP] I think that the autonomy people must have regarding their own health*,* that information*,* and preserving those fundamental patient rights in an important life phase. I think that is important*,* because we are coming from a time when decisions were made about and for patients*,* especially in the final phase of life.” (Quote GP3.1*,* GP interview 1)*	ACP can be confronting and raises negative emotions	*“Well*,* I think we should be open to it*,* if it can be improved. But for me personally*,* I thought some things were very confronting. […] I think*,* one*,* maybe because of my diagnosis. And two*,* I think also because of my age. But I admit that there are things I haven’t considered at all. And then a lot of those questions were difficult for me.” (Quote PT3.1*,* Patient interview 7)*
	Not all materials delivered during the training (conversation guide, template) are always perceived as useful	***Interviewer: “The template to use is the conversation guide without example questions. Did you use this***,*** before or after the conversation?”*** *GP: “No*,* that did not add any dimension that would have been meaningful*,* but which I didn’t already have. […] I only used a piece of what was offered.” (Quote GP3.2*,* Focus group 2)*	Patient supportiveness of ACP	***Interviewer: “And how did that come across to you?”*** *Patient: “I supported it immediately.”* ***Interviewer: “You supported it.”*** *Patient: “Yes*,* because I have a certain opinion about the end of life. Later in my life. And I thought that*,* I was really*,* I won’t say enthusiastic to participate but I did it gladly.” (Quote PT3.2*,* Patient interview 10)*
	GPs were unable to schedule conversations	*“If you already got it down or if it were less important*,* you might say*,* “I’ll just do it quickly and we will see.’ But if you start and it doesn’t go well*,* then you’re better off not doing it.” (Quote GP3.3*,* GP interview 3)*	Patient appraisal of ACP as relevant or not relevant	*“At the moment*,* I don’t need it. And you don’t know how it’ll be a year from now*,* or two years from now*,* or ten years from now.” (Quote PT3.3*,* Patient interview 2)*
Implementation	General satisfaction with the training, but some expectations for more intensive exercise not met	*“For me*,* the interesting part was the discussion and the insight from others. […] Especially the difficulties*,* the limitations*,* how they resolve this*,* sentences to use. Also hearing that others encounter the same obstacles as yourself.” (Quote GP4.1*,* GP interview 2)* ***Interviewer: “I heard Dr. [name] say***,*** more role-play exercises.”*** *GP 1: “Practicing with concrete case examples*,* things you can get stuck on and then tips and tricks to get through that.”* *GP 2: “That’s the advantage of a role-play exercise. You hear each other’s opinions and how someone else would do it*,* you learn a lot from that.” (Quote GP4.2*,* Focus group 1)*	Satisfaction with form/content of workbook, but sometimes difficult to appraise due to limited recall	***Interviewer: “So you have the LEIF-card [pocket card with information about which ADs the person has]***,*** and you’ve also looked at the LEIF-booklet [booklet about different ADs]. Do you think that what we gave you***,*** that booklet***,*** has any added value on top of that?”*** *Patient: “Well yes*,* with a little more explanation about it.”* ***Interviewer: “More explanation***,*** in the LEIF-booklet or ours?”*** *Patient: “In yours it’s more in a language of*,* how do I want…” (Quote PT4.1*,* Patient interview 10)*
	Workbook is a helpful tool for preparation and during conversations	*“But the brochure [referring to workbook] makes the difference*,* then there is more space to do more in one conversation.” (Quote GP4.3*,* GP interview 2).*	Perceived and desired control over decision-making in the ACP process	*“Because at the end of the day we are patients*,* yes*,* well*,* as I say*,* we don’t speak with a full understanding*,* we have to undergo it. I don’t know what needs to happen*,* if suddenly I’m paralyzed*,* just to name something. Yes*,* then it’s necessary to help me and I can’t do anything about that*,* right?” (Quote PT 4.2*,* Patient interview 4)*
	Practical preparation of conversation appointments	***Interviewer: “To implement those conversations in your practice***,*** did you have to make any changes? Or do anything differently?”*** *GP: “No*,* I did that in my free time*,* so it has nothing to do with my practice. My colleagues had little to do with it.”* *GP2: “It was the same for me*,* I also did it on a free afternoon.” (Quote GP4.4*,* Focus group 1)*	Prior experiences with ACP and ADs	*“Um*,* but*,* and we also filled things out a while ago*,* and registered it [with the municipality]. That they can’t reanimate. And also with the LEIF-card [pocket card with information about which ADs the person has].” (Quote PT4.3*,* Patient interview 3)*
	Importance of GP self-efficacy	*“You have to ensure that you don’t do anything wrong by it. If you frighten people… We talked about that quite a bit. How do you convey it properly? What should or shouldn’t you do? What do you avoid?” (Quote GP4.5*,* GP interview 3)*	Prior relationship with the GP	*“Yes*,* and the difficult part*,* is that my actual GP here*,* Dr. [name*,* GP not involved in the study] […] Yes*,* they moved to [city]. And yes*,* that was a little difficult. I can talk to Dr. [GP involved in the study]*,* he was aware of it too*,* but it’s a little different*,* yes.” (Quote PT4.4*,* Patient interview 5)*
	Anticipated interactions with patients	*“The patient was prepared by reviewing the questions and because you had visited. They knew what was coming next.” (Quote GP4.6*,* GP interview 4)*	Experiences with the ACP conversations: -Positive experience -Bidirectional openness between GP and patient -GP asked questions to encourage discussion	***Interviewer: “Did you have the chance to also address what you wanted to discuss during the conversation?”*** *Patient: “Yes*,* I did. I asked personal questions about that care…the person who then has authority over you. I was able to do all of that.”* ***Interviewer: “Did you feel like your GP listened to you and showed understanding for what you brought up?”*** *Patient: “Yes*,* I did.” (Quote PT4.5*,* Patient interview 1)*
	Experiences with the ACP conversations: -Patients did not all use workbook to the same extent, which affects conversation -Themes differ from patient to patient -Difficult for some patients to understand topics	*“With one patient I talked at length about what she would want in terms of care later*,* hospitalizations and the like. With another patient it was primarily about what was important to her in this moment and what she definitely wants to maintain*,* which is contact with her granddaughter. So it differs per patient.” (Quote GP4.7*,* Focus Group 2)*	SDM presence during the conversation	***Interviewer: “Can you tell me about how that conversation went?”*** *Patient: “It lasted about an hour and I thought it was good that my spouse was there as well. She might have had more questions to ask than I did.” (Quote PT4.6*,* Patient interview 9)*
(Intention for) Maintenance	Changes to training for sustainable implementation	***Interviewer: “Dr. [name] is saying a little further on in the master years***,*** but also the GPs who have been working for a bit longer and are interested in refining their skills.”*** *GP: “The basis is the attitude. If you’re focused on […] it all has to happen in those thirty minutes*,* someone who’s really focused on that*,* they won’t get anything out of [a training]… Yeah.” (Quote GP 5.1*,* Focus group 2)*	ACP perceived as completed vs. intention to maintain ACP with the GP	***Interviewer: “And when would you like to talk about it again with Dr. [name]?”*** *Patient: “Well*,* eight days from today she’s coming over.”* ***Interviewer: “Ah yes***,*** so when she comes over again***,*** you’ll talk about it again?”*** *Patient: “Definitely*,* yes.” (Quote PT5.1*,* Patient interview 10)*
	Patient workbook is a useful tool for future practice application	*“I would especially like to keep using the workbook*,* I put the overview for doctors on the computer so I can look at it. I think the booklet is useful*,* I would give that to a patient if they started talking about [ACP] during a consultation.” (Quote GP5.2*,* GP Interview 2)*	Wanting to discuss ACP with other health professionals/specialists	*Patient: “And I would like to also have that conversation with my nephrologist. But yes*,* of course*,* you can’t just do that*,* just demand that from her as a patient […]”* ***Interviewer: “Yes***,*** that asks a bit more planning because their time is more limited? If I understand correctly.”*** *Patient: “Well*,* the thing is*,* you can hardly sit and talk for an hour with the nephrologist […] Just to say what you would want or what your wishes are*,* and to learn more about how*,* what they do*,* at their level.” (Quote PT5.2*,* Patient interview 6)*
	How to plan and conduct ACP efficiently within limited consultation time?	*“I think the added value has primarily been that we made time for it. That’s where there will always be problems. You should have that conversation with patients very often*,* but then we won’t set aside an hour for it. That’s the added value for the patient now: you’re really making time for it and letting them talk. In normal circumstances it’ll rather be: “We’ll talk about it some other time”. That’s my concern.” (Quote GP5.3*,* Focus group 1)*	Plans to revisit ACP conversations when health or quality of life changes	***Interviewer: “Are there moments where you think: at that moment***,*** it would be useful to have that conversation again?”*** *Patient: “I think so. If I’m not doing well*,* I think I’ll need it. I feel good now*,* but you don’t know how long or what… We will see if the medication works. If the moment comes*,* then it’s alright.” (Quote PT5.3*,* Patient interview 9)*
	Interprofessional: Feasibility and desirability of task delegation within the practice	*“I find it difficult to split something like that up. It doesn’t seem pleasant for the patient to first have a conversation with me*,* and then with another colleague.” (Quote GP5.4*,* GP Interview 4)*	Need for more community/media support	*“I think a media campaign could actually help. I think so*,* personally. Because people wouldn’t talk about it*,* and if you encourage people by saying ‘talk about this topic with your GP’*,* or ‘[your GP] may address this soon’*,* without it being…dramatic*,* and without it*,* uh… meaning that everyone is going to be terminally ill. [laughs]” (Quote PT5.4*,* Patient interview 3)*
	System-level: Need for a structured and unified system to document ACP conversations and ensure transfer of information with other clinicians	*“I think there should be more possibilities in our software*,* just like we can fill in other parameters now. That it’s much clearer. Now it’s something separate*,* and where do you have to write that? A document somewhere in the file*,* or scan it*,* because it’s not clear when someone else opens that file. Something very simple*,* a step-by-step plan*,* which is very clearly visible in the file.” (Quote GP5.5*,* Focus group 1)*		

### Reach (Number, proportion, and representativeness of participants)

1570 GPs affiliated with 837 practices were identified during recruitment (Additional File 4). Of these, 1519 were contacted via telephone, email, and/or leaflet. Of 682 GPs who provided a reason for declining participation, the majority (60.6%) cited a lack of time/being too busy. Fifty GPs (3.3% of GPs contacted) expressed interest and agreement to participate; 35/50 (70% of interested GPs) were enrolled and randomized to intervention (*n* = 18) or control (*n* = 17). All enrolled GPs came from unique practices. Reasons for withdrawal prior to randomization included being unable to identify eligible patients for the study and a lack of time.

GPs identified 117 patients for participation, of whom 95 (81.2%) were included. Of 22 patients not included, eight (36.4%) declined or had no interest, and two (9.1%) found the topic too confronting. Baseline characteristics of participants in both groups are shown in Table S2 (Additional File 3).

#### Perceived factors affecting reach

During interviews, GPs gave varying feedback about the ease of finding eligible patients. Some found limitations to the inclusion criteria (Quote GP1.1). The surprise question was deemed useful in place of a strict age cutoff, but was difficult to apply when patients’ possible future health outcomes were unclear (Quote GP1.2). GPs described how some eligible patients primarily consulted a specialist and not the GP, until they approached the terminal phase (Quote GP1.3). Conversely, patients closer to the end of life were those the GP saw regularly. Finally, some GPs described a selection bias, such as choosing patients with whom they felt comfortable discussing ACP (Quote GP1.4).

### Effectiveness (impact of the intervention, including potential negative effects)

For primary effectiveness, we did not find evidence for superiority of the intervention over the control group in improving the patient outcome (ACP engagement) or GP outcome (ACP self-efficacy) [22]. No major adverse events associated with the intervention were reported. Within the complete sample, seven patients died during the trial period, three of whom were in the intervention group.

#### Perceived added value and impact of the intervention

GPs described how the intervention increased their alertness to ACP and its themes in daily practice. This contributed to GPs’ intention to proactively start conversations (Quote GP2.1). Many GPs described how framing ACP around what is important to the patient to live well, gave them a more positive approach (Quote GP2.2) and helped conversations flow logically. GPs felt this was more fulfilling than an advance directive (AD)-driven approach and it helped some GPs feel more confident and supported.

Conversations facilitated an ACP process where GPs learned valuable information about their patients. GPs explained that they documented topics discussed during and after conversations, sometimes in an AD (Quote GP2.3). As a result of the conversations, GPs felt they would be able to better speak up for the patient’s wishes if the patient became incapacitated, and to articulate this to the patient’s family (Quote GP2.4).

GPs perceived that the workbook and conversations helped patients actively contemplate ACP (Quote GP2.5). However, some GPs described no changes (Quote GP2.6) in their own awareness, knowledge or confidence, as they already had previous experience and found a way of having ACP conversations that worked for them. On the other hand, some GPs felt gaining confidence would first require more practice.

Patients expressed that the intervention helped them think about their future health and care wishes (Quote PT2.1). Several patients described how the results of their conversations with the GP were documented and communicated with involved family members (Quote PT2.2). Multiple patients described a positive affect after the conversations with their GP: conversations assuaged worries and made patients feel reassured that their GP would consider their wishes in future care decisions (Quote PT2.3).

### Adoption (Extent of uptake of intervention components by participants)

#### Training

The GP training consisted of an online module and two live, interactive parts. All GPs registered for the online module. Interactive part one was offered in three sessions. Five GPs attended session 1, five session 2, and six session 3. Two GPs received the session in recorded version. The second interactive part was given in two sessions. Seven GPs attended the first session, eight the second, and three received the session in recorded version.

Training materials were emailed to all GPs and were available online throughout the study period. At T1, most GPs indicated using the materials from the training once or twice (33.3%), or monthly (46.7%); two (12.5%) never used the training materials (Table S3, Additional File 3).

#### Workbook

All patients received the workbook from research staff. Of 39 respondents at T1, approximately one-third (30.8%) indicated they had never used the workbook (Table S4, Additional File 3). Seventeen patients returned copies of their workbook by the end of the study period (37.8% of 45 patients retained to T2).

#### Conversations

At their respective T1 assessment, 13/16 GPs (81.3%) reported having had ACP conversations with patients included in the study, and 33/46 patients (71.7%) reported at least one ACP conversation with their GP (Table S5, Additional File 3).

#### Documentation template

All GPs were provided the documentation template in PDF format. GP questionnaire responses at T1 indicated that 8/30 (26.7%) of first conversations and 1/21 (4.8%) of second conversations were documented using the template. Four GPs returned copies of the template by the end of the study period.

#### Perceived factors influencing adoption

During interviews, GPs endorsed the value of ACP as something important for them to do, though many acknowledged that it is a delicate topic which also requires patients to be receptive (Quote GP3.1). This attitude facilitated adoption of the intervention as a whole. GPs varied in the extent of uptake of materials. They described how they read the conversation guide to prepare for conversations. However, some indicated not finding added usefulness for the documentation template or lacking integration with their current means of documentation, and therefore did not use the template (Quote GP3.2). One interviewed GP who had been unable to schedule conversations cited a lack of time, exacerbated by the COVID-19 pandemic, as the main barrier. The lack of time hindered planning and preparation. The GP did not wish to schedule conversations under these constraints (Quote GP3.3).

Patients’ affective reactions to ACP, and attitudes towards ACP, could facilitate adoption or act as a barrier. For some patients, hearing or thinking about ACP was confronting and raised negative emotions, or concerns that their health was declining, which made it more difficult to engage with the topic directly (Quote PT3.1). Some patients acknowledged the benefit of ACP generally but did not want to be “pushed” into it, or did not feel it was relevant for them personally yet. Others were supportive of ACP or felt it couldn’t hurt for them to bring it up (Quote PT3.2). Pertaining to perceived relevance, some patients found ACP personally relevant, e.g. due to older age. However, despite engaging with the intervention by reviewing the workbook and/or having conversations with their GP, some patients nonetheless saw ACP as being for older or more dependent persons, or for those with more acute health concerns, and thus did not feel they needed ACP at the moment (Quote PT3.3).

### Implementation

#### Fidelity to protocol

The protocol for the intervention consists of a researcher-delivered part (giving the workbook to patients, delivering the training and conversation materials to GPs, giving the documentation template to GPs), and a part to be implemented by the participants (GPs having two ACP conversations with each patient, GPs filling out the documentation template).

At T1, 22/31 patients (71%) were reported by the GP to have received two conversations, as specified in the protocol. At the moment of their T1 measurement, 14/46 (30.4%) patients reported having had two or more conversations with their GP (Table S5, Additional File 3). Cross-checking GP and patient reports showed that 9/22 patients for whom GPs reported two conversations, also reported having used their workbook at T1. One patient reported using the workbook, had two conversations by T1 as reported by the GP, and had both conversations documented using the template.

#### Training

Training sessions lasted from 1 h 47 min to 2 h 17 min. In the sessions where GPs conducted practice conversations with model patients, two attending GPs conducted a conversation; other attending GPs gave feedback and participated in group discussions only.

GPs rated the usefulness of and satisfaction with the training and the intervention materials highly (Table S3, Additional File 3). Approximately half (53.3%) did not think the training took too much time. For most GPs (73.4%), the training met or exceeded their expectations. Most (60%) would recommend the training to others, but one-third (33.3%) were unsure.

#### Workbook, conversations, documentation

Conversations with the GP met (77.4%) or exceeded (22.6%) patients’ expectations (Table S4, Additional File 3). Patients largely agreed they received the right amount of information (86.2%), that this information was important to them (84.4%), and that the workbook was easy to understand (80%). Half (56.8%) would also recommend the workbook to others, but approximately one third (32.4%) were unsure.

Conversations were anticipated to take maximally 60 min. GPs reported 29/31 (93.5%) of first conversations and 100% of second conversations lasted up to 60 min. Documentation was primarily done in the electronic medical record (EMR): at T1, GPs reported that 27/30 (90%) of first conversations and 16/21 (76.2%) of second conversations were documented in the EMR (Table S5, Additional File 3).

#### Experiences with implementation

In interviews, most GPs expressed being satisfied with the training. GPs appreciated that the online format eliminated the need for physical transit, found the live session content interesting, and valued hearing how other GPs conducted ACP (Quote GP4.1). GPs who felt the training did not meet expectations, expected a more intensive approach, e.g. with more interaction between GPs than they felt the online format allowed, more exercises including demonstrations by the trainer, and practical guidance such as how to keep ACP conversations on track (Quote GP4.2).

GPs described the patient workbook as a helpful tool (Quote GP4.3) and spontaneously compared it to ADs, which they saw as off-putting to patients. Some GPs used the workbook to structure the conversation or filled it in together with the patient, and found it useful to ensure less of the conversation was forgotten. One GP however felt that the workbook may have been too difficult for some patients to use without guidance.

A lack of time could be a challenge to practical preparations for ACP conversations. Some GPs scheduled conversations during their free time instead of during consultation hours (Quote GP4.4). GPs also emphasized the importance of communication skills, a lack of which made conversations more challenging. They did not want to frighten or offend patients (Quote GP4.5). GPs felt it was a benefit that patients would know what to expect from the conversation; this contributed to how GPs anticipated the interaction with the patients would proceed (Quote GP4.6). GPs expressed that conversations were highly individualized. For example, some patients used the workbook intensively in preparation, but others did not. During appointments, GPs encountered barriers to having in-depth conversations when patients showed a “black or white” view of ACP or did not fully understand the topics. Themes discussed during the ACP conversations varied from patient to patient and GPs adjusted their approach accordingly (Quote GP4.7).

Patients were satisfied with the workbook, but at the moment of interviews with the researchers some had difficulty recalling detailed contents, or had misplaced it. They appreciated that the workbook questions were more general than ADs and valued that it encouraged reflection about living well (Quote PT4.1). Perceived and desired control in decision-making as part of ACP differed between patients. Some felt it was their right and responsibility to talk about their wishes and make decisions themselves. Others relinquished control, e.g. by trusting doctors to make the right decisions. Last, some patients were uncertain if they had control in making decisions about their care or treatment. They questioned to which extent ACP discussions would affect the care they received, or considered themselves laypersons who lacked the knowledge to make decisions about treatment (Quote PT4.2).

Prior patient experiences with ACP may have played a role during implementation. Some patients had already talked about ACP with their GP or had completed ADs (Quote PT4.3). This made ACP easier to talk about or revisit. A prior relationship with the GP was an important facilitator to conversations. For most patients, the GP was a trusted person with whom the patient had a longstanding, positive relationship, creating a secure setting to discuss ACP. Other patients placed more trust in specialist care providers, or did not know the GP well prior to the intervention, which could make conversations more difficult (Quote PT4.4). Patient experiences with the conversations described a feeling of reciprocal openness and equal participation during conversations, which facilitated patient comfort and satisfaction (Quote PT4.5). When a surrogate decision maker (SDM) was present during the conversation, patients experienced this as positive. Some SDMs were already involved in care for the patient and could provide support during conversations. In other cases, the SDM was able to ask questions alongside the patient (Quote PT4.6).

### Maintenance (Intention to sustain the intervention over time)

Of GP respondents, two-thirds (66.7%) indicated high interest in using the intervention materials (workbook, conversation guide, conversation flowchart) in the future (Table S3, Additional File 3). Half of patient questionnaire responses (52.8%) indicated high interest in using the workbook in the future; more than one-quarter (27.8%) indicated low interest (Table S4, Additional File 3).

### Perceived factors affecting intention for Maintenance, and participant recommendations for the future

Some GPs who were interviewed saw potential for sustainable implementation of the training through inclusion in bachelor- or master-level coursework, but also as a refresher for GPs with several years of experience. However, the latter may only draw GPs who already are interested in ACP (Quote GP5.1). Suggestions for the best format and session length for the future depended on preference and learning styles: some GPs preferred fully online modules to review on their own time, without attending live sessions, while others suggested also discussing the theoretical background live. GPs were also interested in continuing to use the workbook in practice; they discussed it with colleagues or created copies for future use. GPs saw it as a helpful tool for patients who signaled wanting to discuss ACP during regular consultations (Quote GP5.2).

GPs foresaw challenges to integrating conversations into future practice. Some were concerned that, while they made time for ACP conversations during the study, they would not be able to continue planning and conducting ACP efficiently within their limited consultation time per patient (Quote GP5.3). Some GPs suggested it would be more feasible to discuss ACP over a longer period of time, addressing smaller “chunks” per consultation. Regarding potential future implementation within an interprofessional team, task delegation in group practices and community health centers was proposed as a supporting factor to maintenance, but each GP would probably still do these conversations with the patients they saw regularly (Quote GP5.4). At the system level, unaddressed needs included a unified system for documenting ACP, for which the current EMR lacked a designated section, leading to discrepancies in how and where documentation is recorded (Quote GP5.5).

Patients differed in their intention to engage with ACP in the future. Some saw ACP as “finished” or wanted to let the topic rest after their conversations with the GP, without specifying when they might return to it. Others said they continued to engage with ACP after the study: through contemplation, talking to loved ones, and planning to talk to their GP (Quote PT5.1). Some patients were also in contact with other health providers and contemplated discussing ACP with them, but worried about demanding too much of their time (Quote PT5.2). When asked when might be a good time to revisit ACP, patients indicated this depended on changes in health and perceived quality of life (Quote PT5.3). To improve the intervention, some patients suggested wanting an addition of community-level support which normalizes ACP, such as media messaging which emphasizes that ACP is also relevant for people who are not terminally ill (Quote PT5.4).

## Discussion

### Main findings

We aimed to better understand the implementation of the complex ACP-GP intervention by assessing how the intervention was delivered and how it was experienced by both GPs and patients. We wanted to gain insight into what worked well and what could be improved for a sustainable implementation in general practice. Therefore, we conducted a mixed-methods process evaluation based on the RE-AIM framework.

We found that GP **reach** to participate in the study was low. We encountered low recruitment, similar to studies recruiting GPs to dementia care and palliative care research [33, 34]. **Effectiveness** of the intervention for the primary outcome was low, as it did not improve patient ACP engagement or GP ACP self-efficacy more than control [22]. In interviews, participants described other impacts of the intervention, discussed below. **Adoption** of the intervention components was variable and GP barriers to adoption overlapped with barriers to recruitment. Adoption of the documentation template was low. Due to this low adoption, the **implementation** domain of fidelity to the full intervention, as described in the protocol, was also low. GPs reported greater fidelity to the prespecified two conversations at T1 than patients. Patients reported especially high satisfaction with the ACP conversations. The intention for **maintenance** was moderate among GPs and patients.

### Interpretation of main findings

#### What might explain findings concerning primary intervention outcomes?

The process evaluation offers possible explanations for why no significant differences were found between intervention and control on the primary outcomes of GP ACP self-efficacy, and patient ACP engagement.

Some GPs explained during interviews that they already felt confident to have ACP conversations before the intervention, due to prior practice experience. These GPs may represent participants who were already engaged and motivated for ACP [35]. Other GPs stated they would need more time to practice and build their confidence. Learning by following courses and exchanging experiences with peers may be one way to improve skills, but gaining experience by conducting ACP in daily practice is an equally important strategy [36]. This, however, may require more time than the three-month follow-up from baseline at which we measured our primary outcome [37], and may be hindered by remaining uncertainties about how to incorporate ACP conversations efficiently into daily practice.

During interviews with patients, we found that patients differed in attitudes towards ACP, and in their desire to be involved in decision-making about their health. Prior literature also suggests that patients may prefer to wait until they feel that ACP is clinically relevant [38, 39], even in cases where current health is poor [39]. Similarly, some patients interviewed for this process evaluation did not assess ACP as relevant at the moment, despite some also being supportive of the concept in general. This may affect “readiness” for talking about/documenting wishes for medical care at the end of life, a domain of the primary patient outcome of our trial (ACP engagement). Thus, attention should be paid to conveying the relevance and usefulness of ACP to all adults, such as from a perspective of holistic care in illness [39, 40]. In the ACP-GP workbook, we included vignettes to show how ACP can apply in many health states, but more directly-engaging preparatory work may be needed to bring this message to patients. A patient and public involvement (PPI) approach [41], e.g. through experience-based co-design [42], from the start of intervention development might have identified ways to more closely match the intervention to patients’ needs and barriers as they relate to ACP engagement.

#### What is the value of the intervention as perceived by GPs and patients?

Our primary outcomes were process-oriented, following theoretical frameworks of behavior change [20, 21, 43]. During interviews, we also asked GPs and patients to describe how they experienced the impact of the intervention. Some described a perceived impact similar to items in the questionnaires, such as patients thinking more about their wishes for future care, and GPs feeling capable to speak up for these wishes on the patient’s behalf. However, GPs and patients also described how engaging in conversations engendered feelings of trust and peace of mind, where patients felt reassured that their GP knew and supported their wishes. This impact aligns with important but under-researched outcomes of ACP within the domain of social, relational, and emotional aspects [37, 44, 45], but was not captured by the questionnaire.

For GPs, a recurring theme in interviews was that the intervention offered a more positive framing of ACP, which includes conversations about what “living well” means to the patient. Compared to AD-driven conversations and AD booklets, which they felt were off-putting to patients, this approach felt more fulfilling to GPs and made ACP easier to bring up proactively. It is possible that centering conversations around how best to maintain patient quality of life, mitigated known GP barriers related to fear of depriving patients of hope [14].

### Implications for practice, policy, and research

Despite satisfaction with the intervention and perceived positive impacts by GPs and patients, implementing the intervention may be challenging due to remaining barriers. A lack of time was a significant barrier to reach, adoption, implementation, and potential maintenance. Given limited available time per consultation, GPs were uncertain how to continue “making time” for ACP. This clinician barrier to ACP is frequently reported [46–50] and prompts reflection about how to implement this intervention, and similar interventions, in practice. Sustainably incorporating the intervention into the GP workflow may require broadening the positive framing introduced by ACP-GP, which was widely appreciated and facilitated conversations, to an approach that takes place over the patient’s life course [51].

The low recruitment of GPs prompts considering to which extent ACP-GP might reach GPs outside a trial setting. In Belgium, ACP is promoted to GPs through modalities including (online) courses about ADs [52], training sessions to local peer-review groups [53], and recently a public health initiative with a website, posters, and flyers [54]. Incorporating components of ACP-GP within these initiatives may improve reach and (sustainable) implementation, such as by offering options to match the diverse preferences for avenues and modalities of the training expressed by GPs during this study. Integrating a quality-of-life-oriented approach in a live training session, can supplement judicial information which is now freely available through the practitioner-facing module of the public health campaign website. This could address GPs’ desire for more hands-on exercise while limiting time investment. Possibilities also include wider distribution of the workbook, which GPs saw as a useful tool for their practice. Being able to use the workbook to spontaneously offer information about ACP to patients who express interest or need, would allow GPs to act when ACP is perceived as most relevant, or to explain the relevance of ACP with the workbook as a supportive tool. This potentially addresses in-practice the barriers related to patients’ perceived relevance of ACP, as described above.

Recent literature emphasizes a holistic approach to ACP involving patients, surrogates, the community, clinicians, health systems, and policy [55]. Situating GPs within this holistic approach can support them in their task of ensuring information transfer with other care providers, who also can be involved in discussing ACP with patients. At the intra-practice level, GPs in group settings interviewed for this process evaluation were open to sharing some ACP tasks, while continuing to leverage the positive impact of their own longitudinal relationship with their own patients. In a Belgian survey study, one third of GPs reported being supported by a practice nurse. Most GPs agreed this collaboration positively impacted GP workload, and that nurses are suitable for providing patient education and health promotion advice [56]. This may offer new avenues for approaching ACP in this setting in the future and contribute to reducing barriers related to a lack of time by streamlining the process. It is, however, essential that the division of responsibilities is clear, and that there is continuity between clinicians [36]. This relates to reflections made by GPs about system-level barriers. GPs considered communication technology essential to facilitating (multidisciplinary) collaboration and follow-up of patients, but their unmet needs at this level were not addressed by offering a documentation template. International literature similarly suggests that documentation systems are not designed to optimize entry of ACP information [15], so standardization and ease of access directly within the EMR [57] should be prioritized [58].

Within the context of divisions of responsibilities for ACP in this holistic view, building patient awareness to ensure timely initiation may also require upstreaming conversations from a medicalized context to the community [59]. Presentations and workshops, media messaging, and sharing experiences with peers may promote awareness of ACP and empower patients to have meaningful conversations about living well, outside of a clinical setting. This can create a foundation for conversations with clinicians, who can support patients in making care goals concrete [60].

### Strengths and limitations

A strength of this process evaluation is its use of RE-AIM, an intuitive and understandable established framework that can address questions beyond quantitative findings of primary effectiveness [28]. A mixed-method design using quantitative questionnaire data, and qualitative data from interviews, helped us understand how and why results occurred [29]. Analyzing GP and patient perspectives allowed us to find interacting notions of impact, and assess how GPs and patients evaluated the intervention. It also lets us identify barriers and facilitators at multiple levels, upholding the complexity approach which informed the development of the intervention.

The study also has limitations. Qualitative data were only collected from the intervention group, so potential factors leading to observed changes in outcomes in the control group are underexplored. We have reflected on a possible Hawthorne effect or increased awareness as a result of study procedures in the primary outcome report; asking control patients how their awareness changed or what other experiences they have had with ACP may have been informative in this regard. Some GPs reported selection bias towards patients with whom they felt comfortable discussing ACP. Additionally, a social desirability bias may affect responses, such as self-reported satisfaction, or during interviews. GPs who dropped out at T1 cited a lack of time to continue. One additional GP was not interviewed for the same reason, but was retained to data collection via questionnaires. An ‘exit interview’ with the GPs who dropped out may have added nuance to the findings. However, we also were able to interview GPs who were retained to data collection but did not have conversations with (all of) their patients. Regarding the RE-AIM domains, data collected during focus groups and interviews focused on the individual level, and questions mainly invited individual perspectives. Additionally, the maintenance domain was based on hypothetical responses, based on current experiences with the intervention. Nevertheless, some reflections about domains such as maintenance also hold implications for broader and longer-term setting-level possibilities, such as the place for ACP training within (continuing) medical education. Finally, recruitment of participants for this study was limited to the Flanders and Brussels regions of Belgium. In Belgium, solo GP practices are still the most frequently-occurring forms of practice. Generalizability of our findings to other regions and countries, such as those working primarily with group clinic settings, may thus be limited.

## Conclusions

Implementing the complex ACP-GP intervention in general practice is feasible, and can be successful. When GPs are able to make time for ACP conversations and conduct these using a positive, rather than AD-driven approach, these conversations can be fulfilling and engender feelings of trust and peace of mind. However, the implementation process is challenging and the sustainability is suboptimal. Our findings will guide future research and recommendations for facilitating and implementing ACP in general practice.

### Electronic supplementary material

Below is the link to the electronic supplementary material.

Supplementary Material 1: Components of the complex ACP-GP intervention. Description: A description of the four components of the intervention

Supplementary Material 2: Topic lists for focus groups and interviews. Description: Instructions and structure for semi-structured focus groups with GPs (also used for individual interviews with GPs), and individual interviews with patients. Translated from Dutch to English.

Supplementary Material 3: Additional tables for the results section. Description: Tables with additional information for the results section. Characteristics of interviewed participants, baseline characteristics of GPs and patients recruited to the cluster-randomized controlled trial, in the intervention and control group, satisfaction, and characteristics of intervention conversations (from satisfaction questionnaire)

Supplementary Material 4: CONSORT flowchart of recruitment and retention. CONSORT flowchart showing the flow of recruitment and retention to the cluster-randomized controlled trial

## Data Availability

The data analyzed for the current study are available from the corresponding author on reasonable request.

## Abbreviations

ACP: Advance care planning
AD: Advance directive
MRC: Medical Research Council
RE-AIM: Reach, Efficacy/Effectiveness, Adoption, Implementation, Maintenance
SDM: Surrogate decision maker
