# Supplementary material for: Experiences with implementing advance care planning (ACP-GP) in Belgian general practice in the context of a cluster RCT: a process evaluation using the RE-AIM framework
Source: BMC Prim Care. 2024 Jul 6;25:247. doi: 10.1186/s12875-024-02510-5 (PMC11227713; doi:10.1186/s12875-024-02510-5)
Supplement: Supplementary file 1 — Supplementary Material 1: Components of the complex ACP-GP intervention. Description: A description of the four components of the intervention [file 12875_2024_2510_MOESM1_ESM.docx]

**Additional File 1. ACP-GP intervention components**

| Component | Description |
| --- | --- |
| 1. GP training | The ACP-GP training was initially developed as a face-to-face training. It was adapted to an online format to accommodate COVID-19 pandemic restrictions in Belgium.  Two interactive, small-group web sessions were provided by two trainers experienced in primary care and communication. Each session lasted approximately 2 hours. GPs received preparatory materials and background information through an e-learning module, which remained available throughout the course of the study. Intervention materials, such as the conversation guide and an example of the patient workbook, were made available in PDF format.  In session 1, GPs discussed their experiences with ACP, fictional case examples and reflection questions, barriers and facilitators to ACP, and video examples. In session 2, GPs practiced intervention-specific ACP conversations with model patients, based on the patient workbook, followed by interactive feedback and discussion. |
| 2. ACP workbook for patients | Patients received an ACP workbook (titled “My Wishes for Future Care”) which highlights the importance of ACP at different stages of health. Patients could use the workbook to reflect on topics such as quality of life, worries about future health or care, preferences for decision-making, and whom they can ask to act as a SDM. |
| 3. Patient-centered ACP discussion with conversation guide. | After the training, GPs were asked to conduct a minimum of 2 ACP conversations with each patient: conversation 1 within two weeks after the training, and conversation 2 within one month after the first conversation. The workbook for patients, and the ACP conversation guide for GPs, structured the conversation. GPs were reimbursed by the research team for the consultations. |
| 4. Documentation of the ACP discussion | GPs received a documentation template, based on the conversation guide, which they can fill in to make note of the outcomes of the ACP discussion. |
