# Supplementary material for: Experiences with implementing advance care planning (ACP-GP) in Belgian general practice in the context of a cluster RCT: a process evaluation using the RE-AIM framework
Source: BMC Prim Care. 2024 Jul 6;25:247. doi: 10.1186/s12875-024-02510-5 (PMC11227713; doi:10.1186/s12875-024-02510-5)
Supplement: Supplementary file 2 — Supplementary Material 2: Topic lists for focus groups and interviews. Description: Instructions and structure for semi-structured focus groups with GPs (also used for individual interviews with GPs), and individual interviews with patients. Translated from Dutch to English. [file 12875_2024_2510_MOESM2_ESM.docx]

Additional File 2. Topic Lists for focus groups and interviews

# 1. Topic list for focus groups and interviews with GPs

| Instructions |
| --- |
| Goal: To gain insight into how participating GPs experienced the intervention (training, follow-up, ACP conversations, documentation, …), to determine if the intervention was applied as described in the protocol, to identify barriers and facilitators that may have influenced implementation, and which aspects of the intervention can/should be adapted to increase future acceptability. |
| Design |
| - Focus group with topic list - Maximum duration: approximately 1 hour 30 minutes - Moderator: one researcher takes on the role of leader/moderator and starts the conversation by asking questions of the GPs. The moderator ensures that everyone has a chance to speak and that the conversation does deviate too far from the question. They also introduce new questions from the topic list. - Observer: for a focus group interview, one or more researchers take on the role of observer. The observer checks the materials, makes notes of the interview, and reviews a summary of the contents with the moderator. - The interviews are recorded and transcribed. |
| Materials |
| - Topic list for moderator and observer - Paper for the observer to take notes - Audio-recording device, tested before the interview - Examples of the materials from the intervention (conversation guide, flowchart, documentation template, patient workbook) that can be presented via screenshare |
| General instructions |
| Moderator   - Review the topic list questions well prior to starting the conversation, and to facilitate the conversation during prolonged silence - Keep the topic list in mind, but allow participants to fully formulate their answers to the questions (do not skip to the next question too quickly) - Begin the interview in a clear and structured way - Provide information about the topic and goal of the interview - Inform participants that an audio recording will be made of the interview - Encourage interaction between participants, summarize what has been discussed, and probe in case of uncertainty - Ensure that everyone has a chance to speak. The moderator may have to invite less-vocal participants to share their experiences or opinion. - Ensure that you as moderator do not participate in discussions or ask suggestive questions. Stay as close as possible to the themes in the topic list, invite participants in a neutral manner to share their experiences or opinion, and stimulate/facilitate the interaction.   Observer   - Pay attention to (non)verbal communication of the participants - Monitor the time and alert the moderator, if necessary, at the 1-hour-15-minute mark (to begin wrapping up the focus group) - Make notes of the content of the discussion. You can ask questions if necessary to facilitate the conversation, but do not take over for the moderator - Make a summary of the most important topics - Evaluate the discussion with the moderator, immediately after the focus group |

| Introduction |  |
| --- | --- |
| - Interviewers (moderator, observer) introduce themselves - Explain the aim of the interview and emphasize that the interview is open to discussing all opinions, questions, and experiences. - Ask participants to turn on their webcam if possible. - Explain that the interview will be recorded and that the transcript, analyses, and results will be processed with respect for confidentiality. - Ask if there are any further questions - Observer: start the audio recording. Briefly verbally indicate that the audio recording is running. | |
| Theme 1: Training | |
| Introduction: At the start of the study period, you were invited to attend a training about ACP communication skills. This training consisted of an online module and two sessions that were delivered live: a session about experiences and barriers, and a session with simulation patients.  We would like to discuss your experiences with and opinions about the training. The training was delivered in the context of the study, but we aim to optimize and implement it more broadly in the future. Your answers can tell us how we might achieve this. | |
| - The training was originally intended as two face-to-face workshops, but had to be delivered online, as an online platform and two web sessions, due to COVID-19 measures.   - What did you think of this approach?   - If you could choose between an online version or face-to-face, what would you choose and why? | |
| - What were your expectations of the training?   - Were your expectations met? Why or why not? | |
| - Whom do you think is the most suitable target group for the training? | |
| - Did the didactic method match your needs?   - If not, which forms of working would better match your practice or needs (e.g. smaller-group discussions, homework exercises, …)? | |
| - Experiences with the training: | |
| - - Which part was most novel or useful to you? What was less useful? | |
| - - Were there any challenges or difficulties during the training? How were these addressed? | |
| - - How much time did you spend on the online module? Did you revisit this after the live sessions? | |
| - - What would you like to see added to the training? What could be left out? | |
| - - Were there questions you wanted to ask or difficulties you wanted to address? If yes, did you receive enough answer/support from the trainer/researcher (during and after the training)? | |
| - Would you recommend the training to colleagues? Why or why not? If not, what should change before you would recommend the training? | |
| Theme 2: ACP conversations (experiences, barriers, facilitators) | |
|  | |
| - After the training, did you feel sufficiently prepared to have the study-specific ACP conversations with your patient?   - If not, what would have helped you feel sufficiently prepared? | |
| - Was it easy or difficult to plan conversations with patients, and why? | |
| - How were the conversations? What did you gain from the conversations and what do you think the patient gained?   - What do you consider a “good” ACP conversation?   - Were you able to achieve this during your conversations with your patients? | |
| - How did you use the conversation materials (conversation guide, flowchart) during the ACP conversations? | |
| - Which themes from the conversation guide/the workbook were most useful to discuss and which was least useful?   - Most or least difficult? | |
| - What did you do differently, in comparison to how you usually conduct ACP conversations with patients in your practice, as a result of the training/the supporting materials?   - Was there a difference for you?   - Was there a difference in the reactions from the patient or the depth of the conversation? | |
| - Did the template for documentation have an added value on top of the existing options (e.g. in the EMR)?   - If yes, what is the added value?   - If no, are there other aspects of documentation you are missing, which were not addressed in the template? | |
| Theme 3: Implementation and maintenance | |
|  | |
| - For patient inclusion, you were asked to identify patients with a chronic, life-limiting illness who meet a 2-year surprise question (you would not be surprised if this patient were to die within 12-24 months).   - Was it easy or difficult for you to identify patients with these criteria?   - How is this similar or different to how you identify patients for ACP in your practice? | |
| - Are there patients we missed in this study due to the inclusion criteria, whom you think would also be helped by the intervention?   - What is needed to reach them? | |
| - The study period is (almost) at an end. Do you still use the intervention (e.g. the conversation materials)?   - If yes, in what way do you use them? | |
| - Do you have suggestions to improve the intervention? | |
| - How would you prefer to implement the intervention in your practice? (e.g. dividing tasks in a group practice, …) | |
| - Are or were any changes necessary to help you use the intervention better in your practice? | |
| Closing/summary questions | |
|  | |
| - We used questionnaires with the aim to understand if/how this intervention improves self-efficacy, knowledge, and attitudes in GPs.   - How do you feel these changed for you?   - What contributed most/least to changes?   - What do you think is necessary to change these outcomes? - Which impact did the intervention have on the way you conduct ACP with your patients?   - What is the added value of the intervention for you?   - What do you think the added value was for your patients? - What is the aim of ACP? Do you think this intervention helps to reach this aim? | |
| Concluding | |
| - We have reached the end of the focus group. Is there anything you would like to add? - Thank you very much for your time, and for sharing your experiences with us.   Observer: indicate verbally that the audio recording is stopped, and immediately stop the recording. | |

# 2. Topic list for the semi-structured interview with the patient and their surrogate decision maker (if present)

| Instructions |
| --- |
| Goal: To gain insight into how participating patients experienced the intervention (workbook, conversations with the GP) to identify barriers and facilitators that may have influenced implementation, and which aspects of the intervention can/should be adapted to increase future acceptability. |
| Design |
| - Semi-structured interview with topic list - Maximum duration: approximately 1 hour (by telephone) - The researcher leads the interview and uses the topic list to ask the patient open-ended questions. - The interviews are recorded and transcribed. |
| Materials |
| - Topic list for the interviewer - Pen and paper for note-taking during the interview - Audio-recorded, tested before start of the interview |
| General instructions |
| - The topics in this list are a guideline, not a checklist. The goal is to explore what the patient’s experiences were and what they recall most about the intervention. If patients cannot recall, questions can be posed about their attitude towards ACP: what would they like to discuss, when, and with whom? - Keep the topic list in mind, but allow participants to fully formulate their answers to the questions (do not skip to the next question too quickly) - Begin the interview in a clear and structured way - Provide information about the topic and goal of the interview - Inform participants that an audio recording will be made of the interview |

| Introduction |
| --- |
| - The interviewer briefly introduces themselves - Explain the aim of the interview and emphasize that the interview is open to discussing all opinions, questions, and experiences. - Emphasize that the interview is intended to allow all opinions, questions, and experiences to be discussed. There are no right or wrong answers, and negative feedback can also be discussed. |
| - Explain that the interview will be recorded and that the transcript, analyses, and results will be processed with respect for confidentiality. |
| - Ask if there are any further questions |
| - Start the audio recording. Briefly verbally indicate that the audio recording is running. |
| General questions |
| - First, I would like to hear what you recall about the study in general: the conversations and the workbook. What did you think of it? - Can you tell me about what you still recall? There are several topics that may have come up. (If necessary, read an example question from the workbook). - Did you learn anything new? What did you learn? - Have you done anything more with everything that came up, after the last conversation? Or would you like to do anything more with it? |
| Questions about the workbook “My Wishes for Future Care” |
| I would like to ask what you thought of the workbook you received at the start of the study.   - Do you recall the workbook? - Did you look at it? Did you write anything in it? - What was your most lasting impression of the workbook? What was your general impression? - Do you see yourself using a workbook like this, outside of a study? What would be needed for you to look at and fill in this workbook, if you had not received it as part of the study?   - Suppose for example, that the workbook was available in the waiting room of the GP’s practice?   - Or if your GP gave it to you after a consultation? - Are there any other workbooks or brochures that you have used? Does the workbook from the study have any added value for you, on top of the workbooks and brochures that already exist? - Are there topics related to ACP that you would like to discuss with family, loved ones, or your GP, but which you did not find in the workbook? |
| Questions about the two ACP conversations |
| Now, I would like to move on to a few questions regarding the conversations your GP had with you about ACP. These were intended to be at least two consultations to which your GP invited you, and during which the workbook may have been discussed.   - Do you recall these conversations? - Can you describe how the conversations went? (If necessary, probe:)   - Did you have the opportunity to raise the topics you most wanted to discuss?   - Did you have the feeling your GP listened to you? Did they show understanding for the things you brought up?   - If you had questions, did you have the chance to ask them? Did you receive an answer to these questions? - What was your general feeling during the conversation? After? - How would you evaluate the conversations yourself, for example with a score out of 10? (If necessary, probe:)   - What did you think was good? What was less good?   - Would you like to plan more conversations like this? Why or why not? At which moment?   - (If surrogate or family was not present at the conversation) Would you consider inviting a loved one or family member to an ACP conversation in the future? Why or why not? |
| Questions for if the patient has limited or no recall of the intervention |
| - Would you like to have a conversation about ACP with your GP in the future? Why or why not? - At which moment would you want such a conversation? - Is your GP someone with whom you would like to discuss ACP? - If you were to talk to your GP about ACP, what would you want to be sure to tell them? What should your GP definitely know?   - (The same questions can also be asked about ACP conversations with a loved one or family member) |
| Concluding |
| - We’ve reached the end of the interview. Is there anything you would like to add? - Thank you very much for your time, and for sharing your experiences with us.   Indicate verbally that the audio recording is stopped, and immediately stop the recording. |
