# Supplementary material for: Experiences with implementing advance care planning (ACP-GP) in Belgian general practice in the context of a cluster RCT: a process evaluation using the RE-AIM framework
Source: BMC Prim Care. 2024 Jul 6;25:247. doi: 10.1186/s12875-024-02510-5 (PMC11227713; doi:10.1186/s12875-024-02510-5)
Supplement: Supplementary file 3 — Supplementary Material 3: Additional tables for the results section. Description: Tables with additional information for the results section. Characteristics of interviewed participants, baseline characteristics of GPs and patients recruited to the cluster-randomized controlled trial, in the intervention and control group, satisfaction, and characteristics of intervention conversations (from satisfaction questionnaire) [file 12875_2024_2510_MOESM3_ESM.docx]

**Table S1. Interview and focus group participant characteristics**

| GPS (n=14) | Focus Group 1 (n=3) | Focus Group 2 (n=2) | Focus Group 3 (n=5) | Individual Interviews (n=4) | Overall |
| --- | --- | --- | --- | --- | --- |
| Age in years, Mean, SD | 55.3 (7.6) | 38 (7.1) | 43.8 (11.9) | 37.5 (10.0) | 43.6 (11.3) |
| Years of practice experience (Mean, SD) | 30.0 (7.9) | 11.5 (9.2) | 16.6 (11.6) | 10.3 (10.2) | 16.9 (11.8) |
|  | **Total (n=)** |  |  |  |  |
| Female sex | 2 | 1 | 1 | 2 | 6 |
| Practice type |  |  |  |  |  |
| Solo | 1 | 1 | 0 | 1 | 3 |
| Group | 2 | 1 | 3 | 3 | 9 |
| Primary care center | 0 | 0 | 1 | 0 | 1 |
| Hospital | 0 | 0 | 0 | 0 | 0 |
| Multiple | 0 | 0 | 1 | 0 | 1 |
| Working as coordinating and advisory physician in a nursing home | 0 | 0 | 1 | 0 | 1 |
| Prior training in ACP |  |  |  |  |  |
| None | 2 | 2 | 2 | 4 | 10 |
| Introductory | 1 | 0 | 3 | 0 | 4 |
| Intensive | 0 | 0 | 0 | 0 | 0 |
| Prior training in palliative care |  |  |  |  |  |
| None | 2 | 2 | 4 | 4 | 12 |
| Introductory | 1 | 0 | 0 | 0 | 1 |
| Intensive | 0 | 0 | 1 | 0 | 1 |
| Patients (n=11) |  | | | | |
| Age in years, Mean, SD | 70.2 (11.2) | | | | |
| Age range | 48-86 | | | | |
|  | **Total (n=)** | | | | |
| Female sex | 4 | | | | |
| Marital status |  | | | | |
| -Married, civil union, or domestic partnership | 6 | | | | |
| -Widow(er) | 3 | | | | |
| -Divorced or single, never married | 2 | | | | |
| Diagnosis |  | | | | |
| Oncological | 4 | | | | |
| Frailty | 5 | | | | |
| Organ failure (renal disease) | 2 | | | | |

**Table S2. Baseline participant characteristics by study arm**

|  | **Control** | **Intervention** |
| --- | --- | --- |
|  | **N(%)** | **N(%)** |
| **GPs (N)** | **17** | **18** |
| **Age ≥37** (sample median; sample range 26-64) | 6 (35.3) | 12 (66.7) |
| **Female gender** | 11 (64.7) | 9 (50) |
| **Years of practice experience ≥9** (sample median; sample range 1-39) | 7 (41.2) | 12 (66.7) |
| **Practice type^a^** |  |  |
| Solo | 4 (23.5) | 4 (22.2) |
| Group | 9 (52.9) | 12 (66.7) |
| Primary care center^b^ | 3 (17.6) | 1 (5.6) |
| Hospital | 0 (0.00) | 0 (0.00) |
| Multiple | 1 (5.9) | 1 (5.6) |
| **Coordinating and advisory physician^c^** | 3 (17.6) | 1 (5.6) |
| **Palliative home care team member** | 1 (5.9) | 0 (0.00) |
| **Prior training in ACP** |  |  |
| None | 14 (82.4) | 13 (72.2) |
| Introductory | 2 (11.8) | 5 (27.8) |
| Intensive | 1 (5.9) | 0 (0.00) |
| **Prior training in palliative care** |  |  |
| None | 11 (64.7) | 11 (61.1) |
| Introductory | 5 (29.4) | 6 (33.3) |
| Intensive | 1 (5.9) | 1 (5.6) |
| **Patients (N)** | **42 ^d^** | **53** |
| **Age≥80** (sample median; sample range 42-95) | 23 (54.8) | 25 (47.2) |
| **Female gender** | 25 (59.5) | 25 (47.2) |
| **Marital status** |  |  |
| Married, civil union, domestic partnership | 17 (40.5) | 28 (52.8) |
| Widow(er) | 17 (40.5) | 20 (37.7) |
| Divorced, or single never married | 8 (19) | 5 (9.4) |
| **Highest educational attainment** |  |  |
| Primary school | 5 (11.9) | 13 (24.5) |
| Secondary school | 29 (69) | 33 (62.3) |
| Post-secondary school | 6 (14.3) | 7 (13.2) |
| None of the above | 2 (4.8) | 0 (0.0) |
| **Person most involved in care** |  |  |
| Spouse or partner | 11 (26.8) | 24 (45.3) |
| Child | 17 (41.5) | 15 (28.3) |
| Other family member | 5 (12.2) | 7 (13.2) |
| Other, not a family member | 7 (17.1) | 6 (11.3) |
| No person identified | 1 (2.4) | 1 (1.9) |
| **Living together with person most involved in care** | 11 (27.5) | 24 (45.3) |
| **Religion** |  |  |
| Religious (Christianity) | 26 (61.9) | 31 (58.5) |
| Not religious | 15 (35.7) | 20 (37.7) |
| Prefer not to say | 1 (2.4) | 2 (3.8) |
| **Advance directives (AD) completed^e^** |  |  |
| AD to refuse medical interventions | 7 (16.7) | 8 (15.1) |
| AD for euthanasia^f^ | 9 (21.4) | 9 (17.0) |
| AD for funerary arrangements | 5 (11.9) | 4 (7.5) |
| AD for organ donation | 1 (2.4) | 3 (5.7) |
| Testament for donating the body to medical   science after death | 1 (2.4) | 1 (1.9) |
| Other directive(s) | 0 (0.0) | 4 (7.5) |
| None | 31 (73.8) | 39 (73.6) |
| Oncological diagnosis | 15 (35.7) | 17 (32.1) |
| ^a^ Belgian GPs are providers of primary care; GPs may work in single-physician (solo) practices, in (sometimes multidisciplinary) group practices with multiple GPs, and in multidisciplinary primary care centers.  ^b^ Primary care setting with a multidisciplinary collaboration, including one or more general practitioners, which is highly accessible and has a low financial threshold.  ^c^ General practitioner, preferably trained in gerontology, who is responsible for the coordination, organization, and continuity of medical care within a nursing home. A coordinating and advisory physician also manages the training of nursing home staff, including in the field of palliative care.  ^d^ Missing values: Person most involved in care n=1; 2 Living together with person most involved in care n=2  ^e^ Multiple responses possible  ^f^ AD for euthanasia in the case of irreversible coma | | |

**Table S3. GP satisfaction questionnaire (T1)**

|  | Number of respondents endorsing option (valid %)^a^ | | | | | | | | | | | |
| --- | --- | --- | --- | --- | --- | --- | --- | --- | --- | --- | --- | --- |
|  | **Low rating (1-3)** | | | | | **Neutral rating (4)** | | | | | **High rating (5-7)** | |
| How useful did you find… |  | | | | |  | | | | |  | |
| the training? | 0 (0.0) | | | | | 1 (6.7) | | | | | 14 (93.3) | |
| the follow-up with the trainers? | 2 (13.3) | | | | | 7 (46.7) | | | | | 6 (40.0) | |
| the intervention materials, to be used during conversations with included patients? | 1 (6.7) | | | | | 1 (6.7) | | | | | 13 (86.7) | |
| How satisfied are you with… |  | | | | |  | | | | |  | |
| the training? | 1 (6.7) | | | | | 2 (13.3) | | | | | 12 (80.0) | |
| the follow-up with the trainers? | 1 (6.7) | | | | | 5 (33.3) | | | | | 9 (60.0) | |
| the intervention materials, to be used during conversations with included patients? | 1 (6.7) | | | | | 1 (6.7) | | | | | 13 (86.7) | |
|  |  | | | |  | | | |  | | | |
| How interested are you to use the materials from the training (e.g. the workbook, conversation guide, conversation flowchart) in the future? | 1 (6.7) | | | | | 4 (26.7) | | | | | 10 (66.7) | |
|  | **Disagree (1-3)** | | | | | **Neutral (4)** | | | | | **Agree (5-7)** | |
| The information I received during the intervention was important to me. | 0 (0.0) | | | | | 5 (33.3) | | | | | 10 (66.7) | |
| I felt uncomfortable with the information that was brought up during the intervention. | 14 (93.3) | | | | | 1 (6.7) | | | | | 0 (0.0) | |
| The intervention training took too much time. | 8 (53.3) | | | | | 3 (20.0) | | | | | 4 (26.7) | |
| The intervention (e.g. materials and information from the training) was easy to understand. | 1 (6.7) | | | | | 1 (6.7) | | | | | 13 (86.7) | |
| I can use the information from the intervention in my daily practice. | 0 (0.0) | | | | | 3 (20.0) | | | | | 12 (80.0) | |
|  | | | **It did not meet my expectations** | | | | **It met my expectations** | | | **It exceeded my expectations** | | |
| To what extent did the training meet your expectations? | 4 (26.7) | | | | | | 10 (66.7) | | | 1 (6.7) | | |
|  | **Not enough**  **information** | | | | | | | **The right amount of information** | | **Too much information** | | |
| What did you think of the amount of information you received during the training? | 4 (26.7) | | | | | | | 11 (73.3) | | 0 (0.0) | | |
|  | | **Never** | | **Once or twice in total** | | **Monthly** | | | **Weekly** | | **Daily** | |
| How often did you use the materials from the training? | | 2 (13.3) | | 5 (33.3) | | 7 (46.7) | | | 1 (6.7) | | 0 (0.0) | |
|  | **No** | | | | | | | **Not sure** | | | | **Yes** |
| Would you recommend the training to other GPs? | 1 (6.7) | | | | | | | 5 (33.3) | | | | 9 (60.0) |

_a. Missing data: One GP satisfaction questionnaire missing in full_

**Table S4. Patient satisfaction questionnaire (T1)**

|  | Number of respondents endorsing option (valid %) | | | | |
| --- | --- | --- | --- | --- | --- |
|  | **Low rating (1-3)** | | **Neutral rating (4)** | | **High rating (5-7)** |
| How useful did you find the conversations with your GP, based on the workbook? | 1 (3.2) | | 3 (9.7) | | 27 (87.1) |
| How satisfied are you with the conversations with your GP, based on the workbook? | 0 (0.0) | | 1 (3.2) | | 30 (96.8) |
| How interested are you to use the materials from the study (e.g. the workbook) in the future? | 10 (27.8) | | 7 (19.4) | | 19 (52.8) |
|  | **Disagree (1-3)** | | **Neutral (4)** | | **Agree (5-7)** |
| The information I received during the conversations with my GP was important to me. | 2 (6.3) | | 3 (9.4) | | 27 (84.4) |
| I felt uncomfortable with the information that was brought up during the conversations with my GP. | 23 (69.7) | | 4 (12.1) | | 6 (18.2) |
| The conversations with my GP took too much time. | 30 (93.8) | | 1 (3.1) | | 1 (3.1) |
| The workbook was easy to understand. | 3 (8.6) | | 4 (11.4) | | 28 (80.0) |
| I can use the information from the conversations with my GP, in my daily life. | 7 (21.9) | | 6 (18.8) | | 19 (59.4) |
|  | **They did not meet my expectations** | | **They met my expectations** | | **They exceeded my expectations** |
| To what extent did the conversations with your GP, based on the workbook, meet your expectations? | 0 (0.0) | | 24 (77.4) | | 7 (22.6) |
|  | **Not enough information** | | **The right amount of information** | | **Too much information** |
| What did you think of the amount of information you received in the workbook and during the conversations? | 2 (6.9) | | 25 (86.2) | | 2 (6.9) |
|  | **Never** | **Once or twice in total** | **Monthly** | **Weekly** | **Daily** |
| How often did you use the workbook from the intervention? | 12 (30.8) | 19 (48.7) | 5 (12.8) | 3 (7.7) | 0 (0.00) |
|  | **No** | | **Not sure** | | **Yes** |
| Would you recommend the workbook to other patients? | 4 (10.8) | | 12 (32.4) | | 21 (56.8) |

**Table S5. ACP conversations, length, and documentation, from questionnaire (T1)**

| GP-reported |  |  |  |  |
| --- | --- | --- | --- | --- |
|  | **N** | **% of valid responses (/16)** |  |  |
| GPs who conducted ACP conversations with patients involved in the study | 13 | 81.3 |  |  |
|  | **N** | **% of valid responses (/31)** |  |  |
| Number of patients who received… |  |  |  |  |
| One conversation | 9 | 29.0 |  |  |
| Two or more conversations | 22 | 71.0 |  |  |
|  | **Conversation 1 (N)** | **% of valid responses (/31)** | **Conversation 2 (N)** | **% of valid responses (/22)** |
| Length of conversation |  |  |  |  |
| <15 minutes | 1 | 3.2 | 9 | 40.9 |
| 15-30 minutes | 14 | 45.2 | 8 | 30.4 |
| 31-60 minutes | 14 | 45.2 | 5 | 22.7 |
| >60 minutes | 2 | 6.5 | 0 | 0.0 |
|  |  | **% of valid responses (/30)^a^** |  | **% of valid responses (/21)^a^** |
| Documented using^b^ |  |  |  |  |
| Intervention template | 8 | 26.7 | 1 | 4.8 |
| Patient EMR | 27 | 90.0 | 16 | 76.2 |
| Advance directive | 2 | 6.7 | 6 | 20.0 |
| Documented elsewhere | 3 | 10.0 | 3 | 10.0 |
| No modality of documentation reported | 1 | 3.3 | 4 | 19.0 |
|  |  |  |  |  |
| Patient-reported |  |  |  |  |
| Number of ACP conversations with GP | **N** | **% of valid responses (/46)** |  |  |
| One conversation | 19 | 41.3 |  |  |
| Two or more conversations | 14 | 30.4 |  |  |

^a. One questionnaire about conversations incomplete^

^b. Multiple answers possible^
