# Supplementary figures and images for: Experiences with implementing advance care planning (ACP-GP) in Belgian general practice in the context of a cluster RCT: a process evaluation using the RE-AIM framework
Source: BMC Prim Care. 2024 Jul 6;25:247. doi: 10.1186/s12875-024-02510-5 (PMC11227713; doi:10.1186/s12875-024-02510-5)

Additional File 4. CONSORT chart of recruitment and retention

**
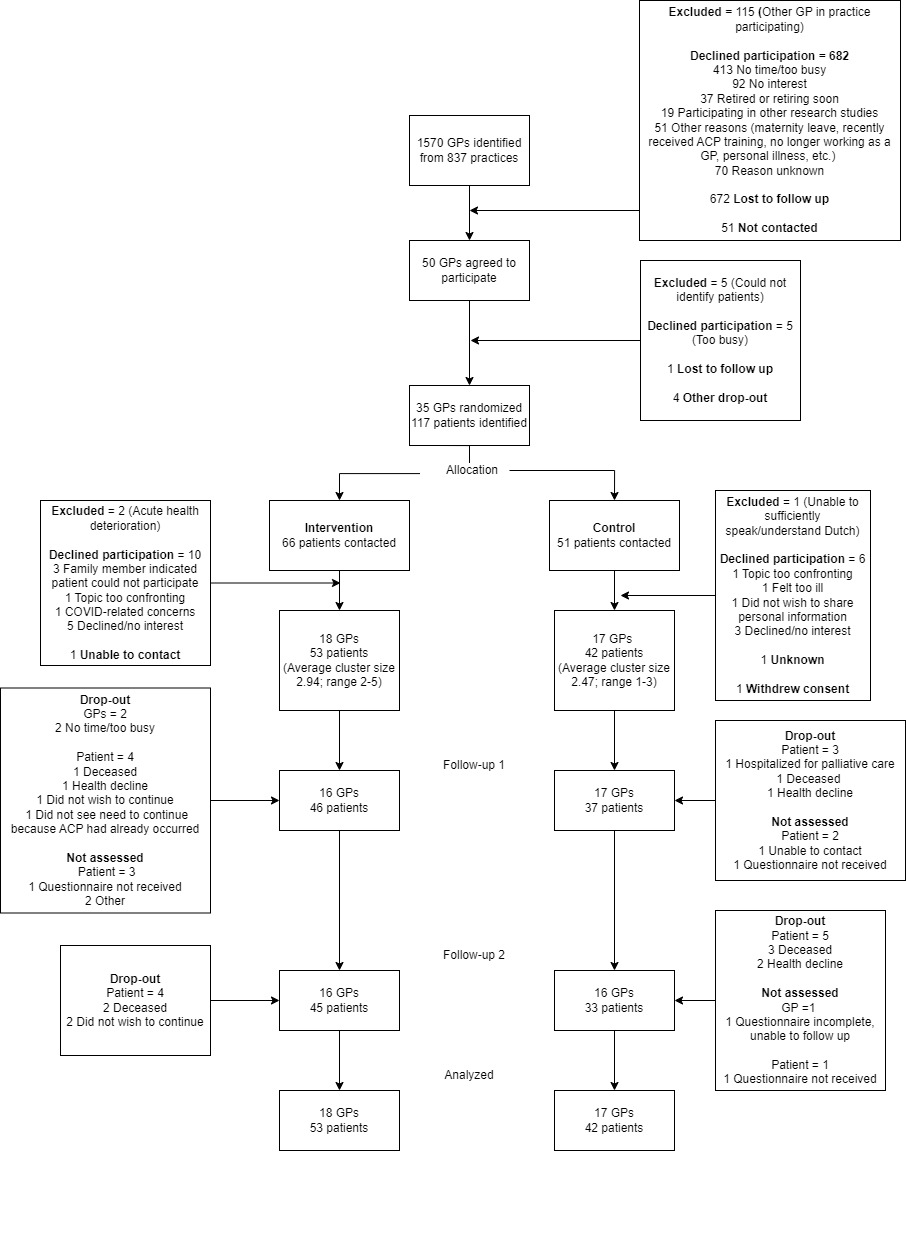
**

Supplement: Supplementary file 4 — Supplementary Material 4: CONSORT flowchart of recruitment and retention. CONSORT flowchart showing the flow of recruitment and retention to the cluster-randomized controlled trial [file 12875_2024_2510_MOESM4_ESM.docx]
